# NEDD1-S411 phosphorylation plays a critical function in the coordination of microtubule nucleation during mitosis

**DOI:** 10.1242/bio.059474

**Published:** 2022-11-17

**Authors:** Krystal Timón Pérez, Jacopo Scrofani, Isabelle Vernos

**Affiliations:** ^1^ Quantitative Cell Biology Program, Centre for Genomic Regulation (CRG), The Barcelona Institute of Science and Technology, Barcelona 08003, Spain; ^2^ Universitat Pompeu Fabra (UPF), Barcelona 08003, Spain; ^3^ ICREA, Pg. Lluis Companys 23, Barcelona 08010, Spain

**Keywords:** Spindle, Microtubule nucleation, NEDD1 phosphorylation, Centrosome, RanGTP, Microtubule branching

## Abstract

During mitosis, spindle assembly relies on centrosomal and acentrosomal microtubule nucleation pathways that all require the γ-Tubulin Ring Complex (γ-TuRC) and its adaptor protein NEDD1. The activity of these different pathways needs to be coordinated to ensure bipolar spindle assembly ( Cavazza et al., 2016) but the underlying mechanism is still unclear. Previous studies have identified three sites in NEDD1 (S377, S405 and S411) that when phosphorylated drive MT nucleation at the centrosomes, around the chromosomes and on pre-existing MTs respectively ( Lüders et al., 2006; Pinyol et al., 2013; Sdelci et al., 2012). Here we aimed at getting additional insights into the mechanism that coordinates the different MT nucleation pathways in dividing cells using a collection of HeLa stable inducible cell lines expressing NEDD1 phospho-variants at these three sites and Xenopus egg extracts. Our results provide further support for the essential role of phosphorylation at the three residues. Moreover, we directly demonstrate that S411 phosphorylation is essential for MT branching using TIRF microscopy in Xenopus egg extracts and we show that it plays a crucial role in ensuring the balance between centrosome and chromosome-dependent MT nucleation required for bipolar spindle assembly in mitotic cells.

## Teaser

**Summary:** The regulation of NEDD1 phosphorylation at S411 is essential for the coordination of the centrosomal and acentrosomal microtubule nucleation pathways and spindle assembly in mitotic cells.

## INTRODUCTION

In higher eukaryotic cells, MT nucleation requires the γ-tubulin ring complex (γ-TuRC), a multi subunit protein complex constituted by multiple copies of γ-tubulin and a number of associated components called gamma tubulin complex proteins (GCPs; Kollman et al., 2011; Wieczorek et al., 2020; Zheng et al., 1995). Although there is an excess of γ-TuRC in the cell, only a fraction drives the nucleation of MTs. The mechanism underlying γ-TuRC activation is still not entirely clear but it may involve conformational changes and/or additional components such as the protein NEDD1. A complex spatial and temporal regulation of MT nucleation occurs during mitosis. Indeed, spindle assembly relies on γ-TuRC/NEDD1-dependent centrosomal and acentrosomal MT nucleation pathways (Meunier and Vernos, 2012).

In most animal cells entering mitosis, the active recruitment of γ-TuRCs to the centrosome occurs before nuclear envelope breakdown in a NEDD1-dependent manner and drives the increase of MT assembly from the centrosome (Mahoney et al., 2006; Piehl et al., 2004). After nuclear envelope breakdown, two additional MT nucleation pathways are activated. One of them is driven by the small GTPase Ran that in its GTP bound form (RanGTP) drives the release of nuclear localization signal (NLS) containing proteins from karyopherins around the chromosomes (Clarke and Zhang, 2008). Some of them drive MT nucleation in a γ-TuRC/NEDD1-dependent manner (Scrofani et al., 2015). In addition, a MT-based MT nucleation pathway involving the multi-subunit Augmin and the γ-TuRC/NEDD1 complexes promotes MT branching and amplification (Clausen and Ribbeck, 2007; Goshima et al., 2008; Petry et al., 2013).

NEDD1 is highly phosphorylated in mitosis (Gomez-Ferreria et al., 2012; Gunawardane et al., 2003; Johmura et al., 2011; Lüders et al., 2006; Manning et al., 2010; Zhang et al., 2009). Previous studies pointed at NEDD1 phosphorylation as a key potential mechanism for the specific control of MT nucleation through the different pathways. Interestingly, single phosphorylation events on different residues were found to control each of the three MT nucleation pathways in mitotic cells: S377 phosphorylation by Nek9 is essential for the recruitment of γ-tubulin to the centrosome in prometaphase and thereby for centrosome-dependent MT nucleation (Sdelci et al., 2012), S405 phosphorylation by Aurora-A is essential for RanGTP/chromosome-dependent MT nucleation (Pinyol et al., 2013; Scrofani et al., 2015) and S411 (S418 in Lüders et al., 2006) phosphorylation by Cdk1 for MT-dependent MT amplification (Lüders et al., 2006; Johmura et al., 2011). These data suggested that NEDD1 phospho-regulation could provide a mechanism for the coordinated activation of MT nucleation during mitosis.

Combining work in HeLa cells and Xenopus *laevis* egg extracts we aimed at getting further insights into the mechanism that coordinates the different MT nucleation pathways in dividing cells by targeting the three specific essential NEDD1 phospho-sites (S377, S405 and S411) alone and in combination.

Our data confirm the strict requirement for NEDD1 phosphorylation at each of the three sites for spindle assembly and reveal a key role for the regulation of NEDD1 phosphorylation at S411 for the coordination of MT nucleation at the centrosome and around the chromosomes during mitosis.

## RESULTS

### A system to study the role of MT nucleation in spindle assembly by targeting NEDD1 phosphorylation

**Fig. 1.**
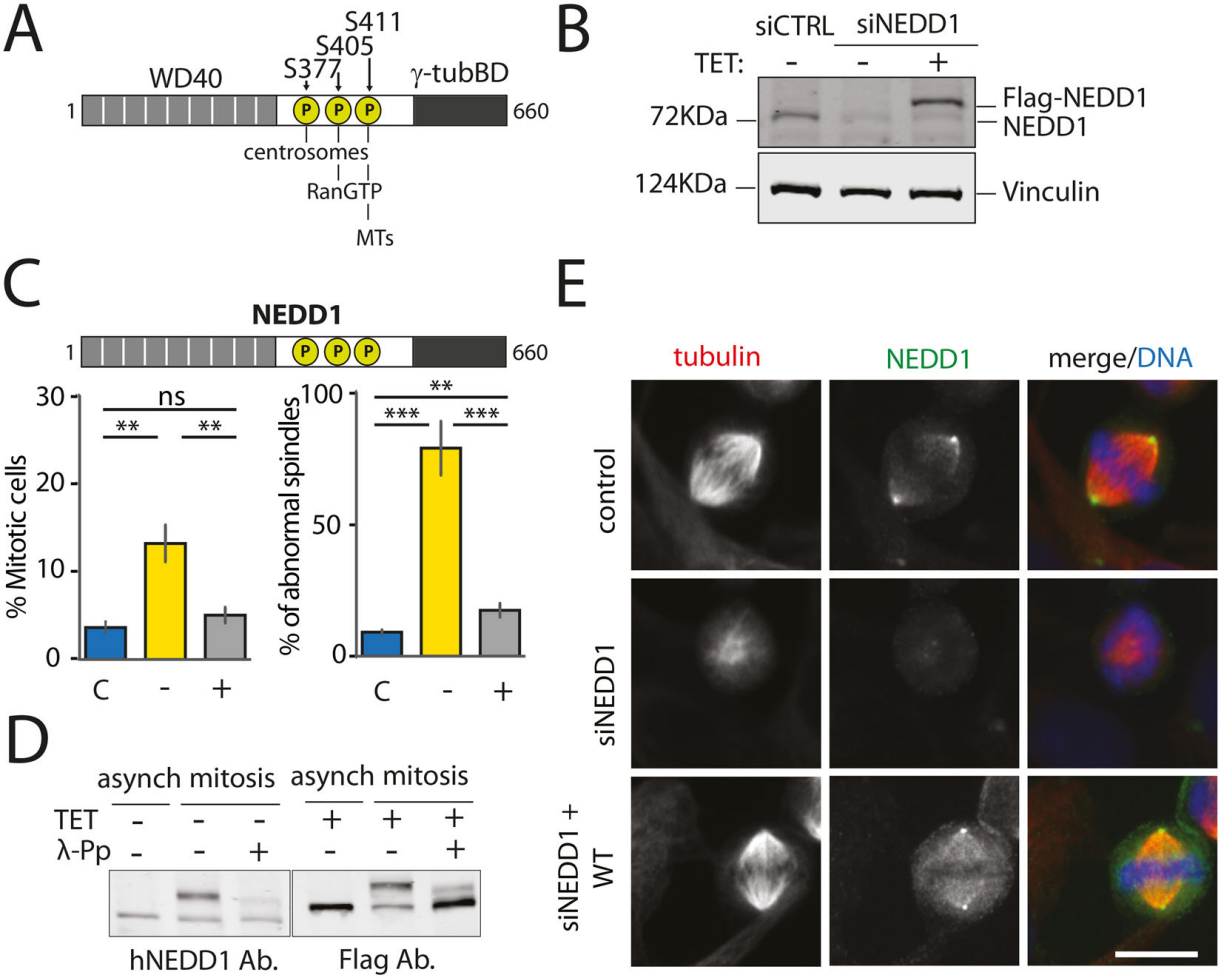
**Inducible expression of Flag-NEDD1 fully rescues spindle assembly and mitotic progression upon NEDD1 silencing in a stable HeLa cell line.** (A) Schematic representation of NEDD1 showing the positions of S377, S405 and S411 and the relevance of their phosphorylation for centrosomal, chromosomal and MT-based MT nucleation. The three sites fall into a regulatory domain placed between the N-terminal WD40 domain and a C-terminal γ-tubulin binding domain. (B) Western blots showing NEDD1 protein levels in control (siCTRL) and NEDD1-silenced (siNEDD1) HeLa cells expressing Flag-NEDD1 under tetracycline control (+TET). The anti-human NEDD1 antibody detects both the endogenous and ectopically expressed Flag-NEDD1 proteins. The vinculin band was used as a loading control. (C) Quantifications made on HeLa cells expressing Flag-NEDD1 under tetracycline control showing the percentage of mitotic cells and abnormal spindles in the different experimental conditions. Blue bars represent control siRNA cells not induced with tetracycline (C); yellow bars are cells transfected with NEDD1 siRNA and not induced with tetracycline (–); grey bars correspond to cells transfected with NEDD1 siRNA, treated with tetracycline and expressing Flag-NEDD1 (+). More than 1000 cells were monitored to calculate the percentage of mitotic cells. More than 200 cells were monitored to quantify the spindle phenotypes. The graph represents the average from three independent experiments. Error bars are standard deviation. ns: not significant; **P<0.01; ***P<0,001. (D) Western blot analysis showing endogenous and ectopically expressed Flag-NEDD1 (+TET) in asynchronous and mitotic HeLa cells. Samples were incubated or not with λ-phosphatase (+λ-Pp). The anti-human NEDD1 antibody detects the endogenous NEDD1. The anti-flag antibody detects Flag-NEDD1 WT. (E) Immunofluorescence of control and NEDD1-silenced (siNEDD1) HeLa cells expressing Flag-NEDD1. DNA is in blue, tubulin is in red. The anti-hNEDD1 antibody recognizes the endogenous and ectopically expressed proteins (green). Scale bar: 10 µm.

To study the contribution of the different MT nucleation pathways in spindle assembly, we aimed at targeting each pathway alone or in combination through the expression of different phosphorylation variants of NEDD1 on S377, S405 and S411 (Fig. 1A). To first establish a robust experimental system, we generated a stable HeLa cell line for inducible expression of siRNA-resistant wild-type Flag-NEDD1. As previously reported, reduction of endogenous NEDD1 levels by siRNA was effective and silenced cells arrested in mitosis with severe spindle defects (Fig. 1B) (Pinyol et al., 2013; Sdelci et al., 2012). Expression of exogenous wild type Flag-NEDD1 in the silenced cells at close to endogenous concentration fully rescued spindle assembly and mitotic progression (Fig. 1C). Consistently, Flag-NEDD1 was highly phosphorylated in mitosis (Fig. 1D) and localized to the centrosomes and the spindle MTs like the endogenous protein (Fig. 1E) (Haren et al., 2009; Lüders et al., 2006; Zhang et al., 2009). This approach therefore provided a robust experimental system to address the role and integration of the different MT nucleation pathways in spindle assembly by expressing NEDD1 phospho-variants on S377, S405 and S411 in silenced cells.

### Triple NEDD1 S377/S405/S411 phospho-mimetic mutant does not support spindle assembly

**Fig. 2.**
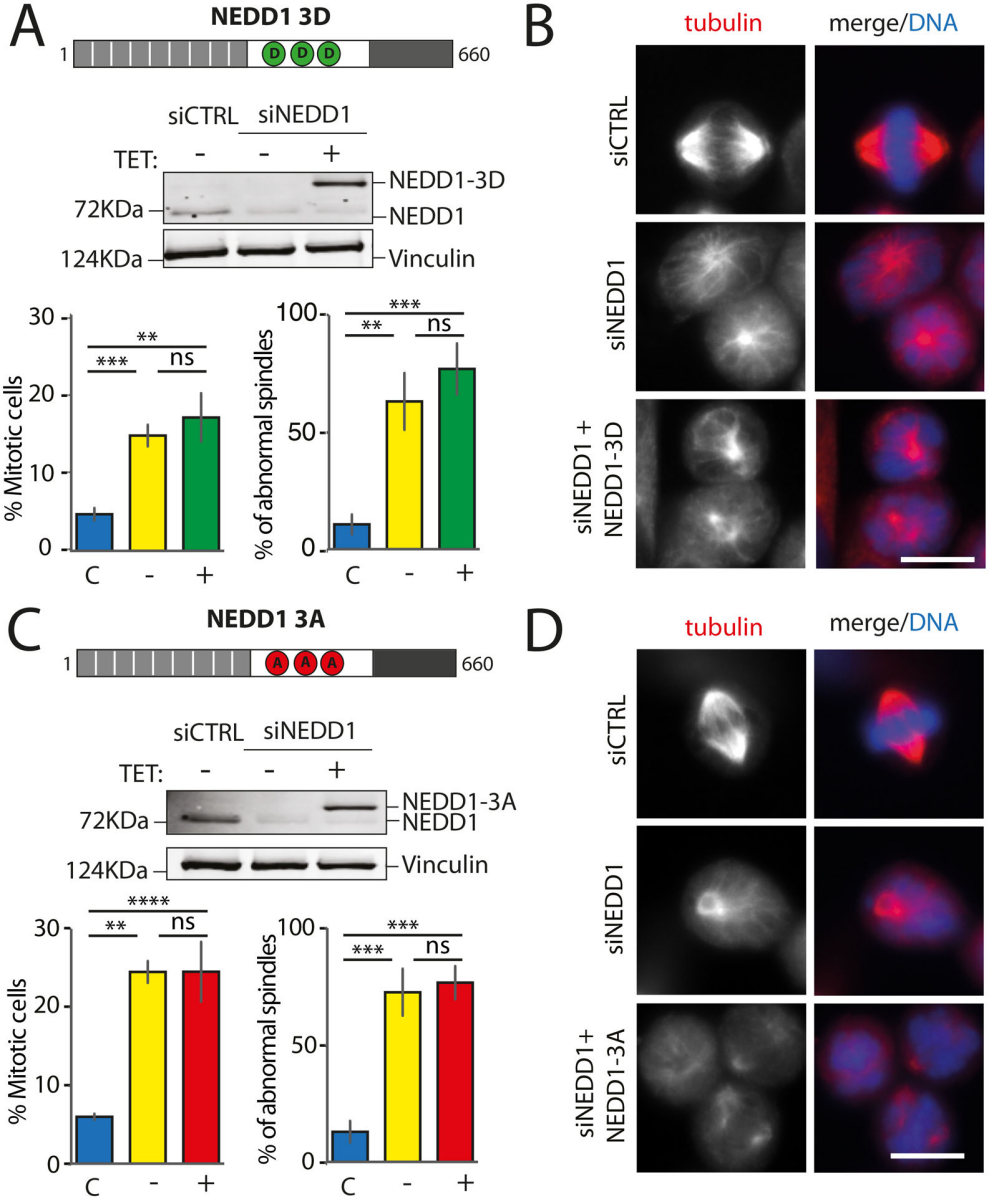
**Mitotic phenotypes in NEDD1 silenced HeLa cells upon expression of NEDD1 triple phospho-variants.** (A,C) Upper panels: western blots showing NEDD1 protein levels in control (siCTRL) and NEDD1-silenced (siNEDD1) HeLa cell lines expressing Flag-NEDD1 phospho-variants (as indicated) under tetracycline control (+TET). The anti-human NEDD1 antibody detects both endogenous and ectopically expressed Flag-NEDD1 proteins. The vinculin band was used as a loading control. Lower panels: quantifications of the percentage of mitotic cells and abnormal spindles in HeLa cells in the different experimental conditions. Blue bars correspond to control cells not induced with tetracycline (C); yellow bars are NEDD1 silenced cells not induced with tetracycline (−); green and red bars correspond to NEDD1 silenced cells induced with tetracycline that express respectively the phospho-mimetic Flag-NEDD1-3D variant or phospho-null Flag-NEDD1-3A, as indicated (+). More than 1000 cells were monitored to calculate the percentage of mitotic cells and more than 200 to quantify the spindle phenotypes. The graphs represent the average from three independent experiments. Error bars are standard deviation. ns: not significant; **P<0,01; ***P<0,001; ****P<0,0001. (B,D) Immunofluorescence of control and NEDD1-silenced (siNEDD1) HeLa cells expressing or not Flag-NEDD1 3D or Flag-NEDD1 3D, as indicated. DNA is in blue and tubulin is in red. Scale bar: 10 µm.

Previous studies have shown that three mitotic kinases (Nek9, Aurora A and Cdk1) phosphorylate NEDD1 on S377, S405 and S411 respectively (Lüders et al., 2006; Pinyol et al., 2013; Scrofani et al., 2015; Sdelci et al., 2012). Since the three kinases localize to the centrosome in G2/M, we decided to test whether the simultaneous phosphorylation of NEDD1 at the three sites could fulfill all the requirements for triggering MT nucleation from the centrosomes, the chromosomes and the MTs. We generated a form of NEDD1 in which all three residues were replaced by phospho-mimetic amino acids Flag-NEDD1[S377D; S405D; S411D] and established a stable HeLa cell line for inducible expression of the triple mutant (Flag-NEDD1-3D). In contrast to Flag-NEDD1, Flag-NEDD1-3D expressed at close to endogenous concentrations in silenced cells did not rescue spindle assembly nor mitotic progression (Fig. 2A). Immunofluorescence analysis revealed that the Flag-NEDD1-3D expressing cells arrested in mitosis with highly aberrant MT assemblies that were reminiscent of those observed in silenced cells (Fig. 2B). Similar results were obtained with another stable inducible cell line expressing the triple phospho-null variant Flag-NEDD1-3A under similar experimental conditions (Fig. 2C and D). Altogether, our data strongly indicated a lack of function for phospho-mimetic Flag-NEDD1-3D, suggesting more complex requirements for the regulation of NEDD1 phosphorylation and its activity in driving the different MT nucleation pathways in mitosis.

### NEDD1 phosphorylations at S377 and S405 are essential for spindle assembly during mitosis

The lack of function of Flag-NEDD1-3D was however somehow unexpected and raised questions about the consequences of the simultaneous substitution of the three residues. We therefore aimed at getting a complete characterization of all the single phospho-variants. Indeed, only Flag-NEDD1-S405D had been previously shown to fully rescue spindle assembly in silenced cells (Pinyol et al., 2013). We generated six stable inducible HeLa cell lines for the individual expression of the Flag-NEDD1 phospho-variants (phospho-null and phospho-mimetic) for each of the three sites: S377, S405 and S411.

**Fig. 3.**
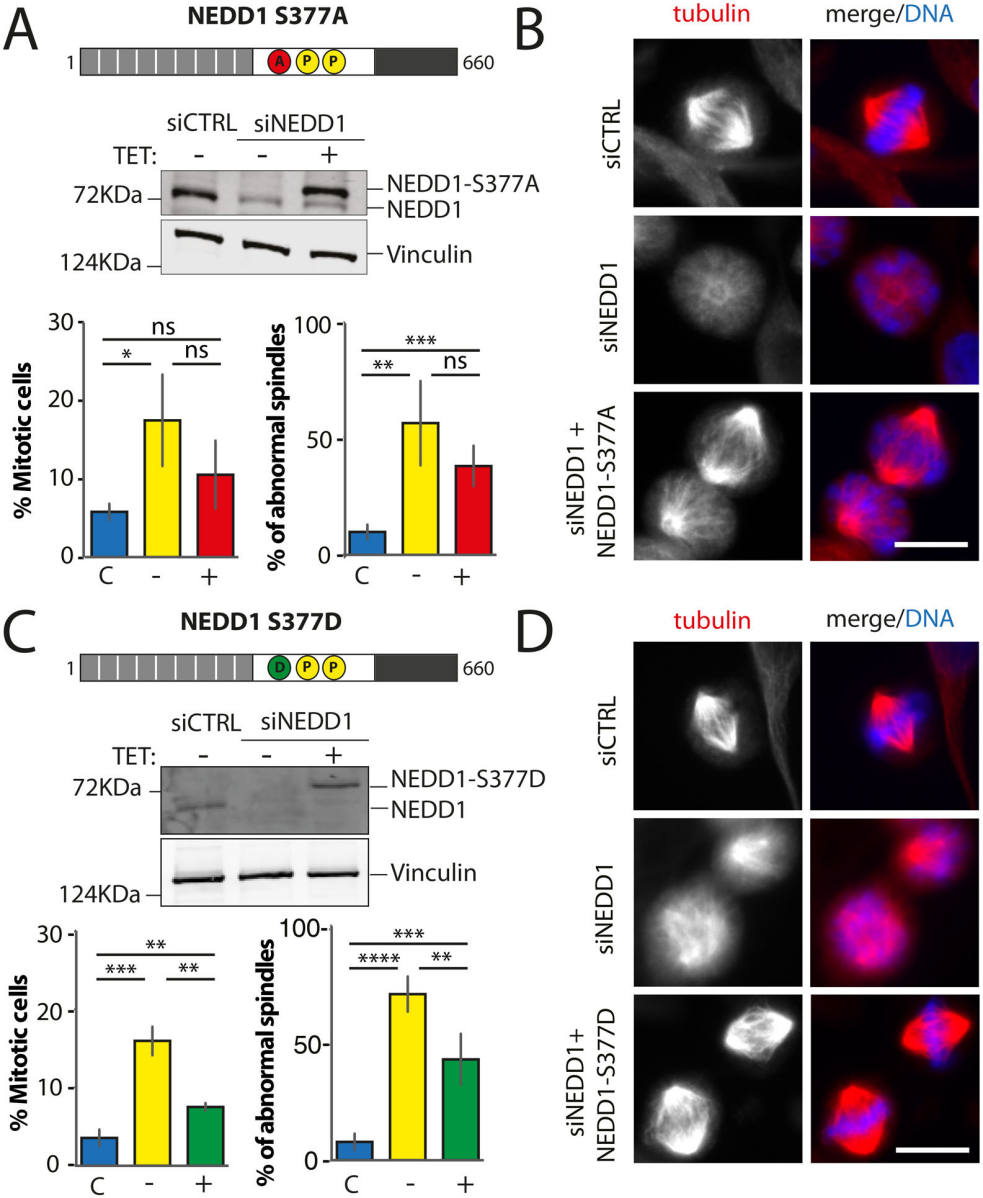
**Mitotic phenotypes in NEDD1 silenced HeLa cells upon expression of NEDD1 S377 phospho-variants.** (A,C) Upper panels: western blots showing NEDD1 protein levels in control (siCTRL) and NEDD1-silenced (siNEDD1) HeLa cell lines expressing or not Flag-NEDD1 phospho-variants at S377, as indicated (+TET). The anti-human NEDD1 antibody detects both the endogenous and ectopically expressed Flag-NEDD1 proteins. The vinculin band was used as a loading control. Lower panels: quantifications of the percentages of mitotic cells and abnormal spindles in HeLa cells in the different experimental conditions. Blue bars correspond to control cells not induced with tetracycline (C); yellow bars are NEDD1 silenced cells not induced with tetracycline (–); green and red bars correspond to NEDD1 silenced cells induced with tetracycline that express respectively the phospho-null Flag-NEDD1-S377A or phospho-mimetic Flag-NEDD1-S377D variants, as indicated (+). More than 1000 cells were monitored to calculate the percentage of mitotic cells and more than 200 to quantify the spindle phenotypes. The graphs represent the average from three independent experiments. Error bars are standard deviation. ns: not significant; **P<0,01; ***P<0,001; ****P<0,0001. (B,D) Immunofluorescence of control (siCTRL) and NEDD1-silenced (siNEDD1) HeLa cell lines expressing Flag-NEDD1-S377A or Flag-NEDD1-S377D. DNA is in blue and tubulin is in red. Scale bar: 10 µm.

**Fig. 4.**
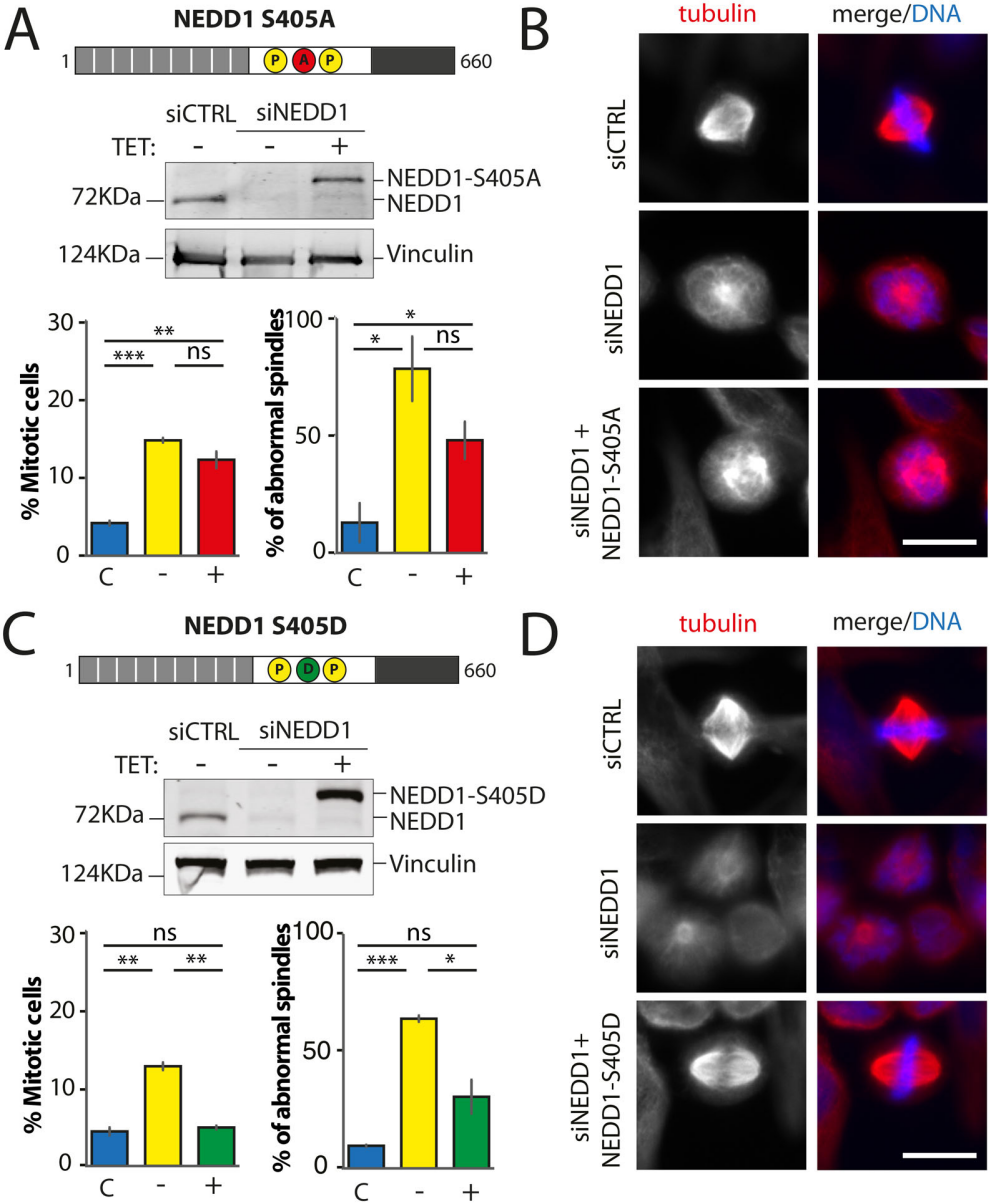
**Mitotic phenotypes in NEDD1 silenced HeLa cells upon expression of NEDD1 S405 phospho-variants.** (A,C) Upper panels: western blots showing NEDD1 protein levels in control (siCTRL) and NEDD1-silenced (siNEDD1) HeLa cell lines expressing or not Flag-NEDD1 phospho-variants at S405, as indicated (+TET). The anti-human NEDD1 antibody detects both the endogenous and ectopically expressed Flag-NEDD1 proteins. The vinculin band was used as a loading control. Lower panels: quantifications of the percentages of mitotic cells and abnormal spindles in HeLa cells in the different experimental conditions. Blue bars correspond to control cells not induced with tetracycline (C); yellow bars are NEDD1 silenced cells not induced with tetracycline (–); red and green bars correspond to NEDD1 silenced cells induced with tetracycline that express respectively the phospho-null Flag-NEDD1-S405A or phospho-mimetic Flag-NEDD1-S405D variants, as indicated (+). More than 1000 cells were monitored to calculate the percentage of mitotic cells and more than 200 to quantify the spindle phenotypes. The graphs represent the average from two independent experiments. Error bars are standard deviation. ns: not significant; *P<0,05; **P<0,01; ***P<0,001. (B,D) Immunofluorescence of control (siCTRL) and NEDD1-silenced (siNEDD1) HeLa cells expressing Flag-NEDD1 S405A or Flag-NEDD1-S405D. DNA is in blue and tubulin is in red. Scale bar: 10 µm.

**Fig. 5.**
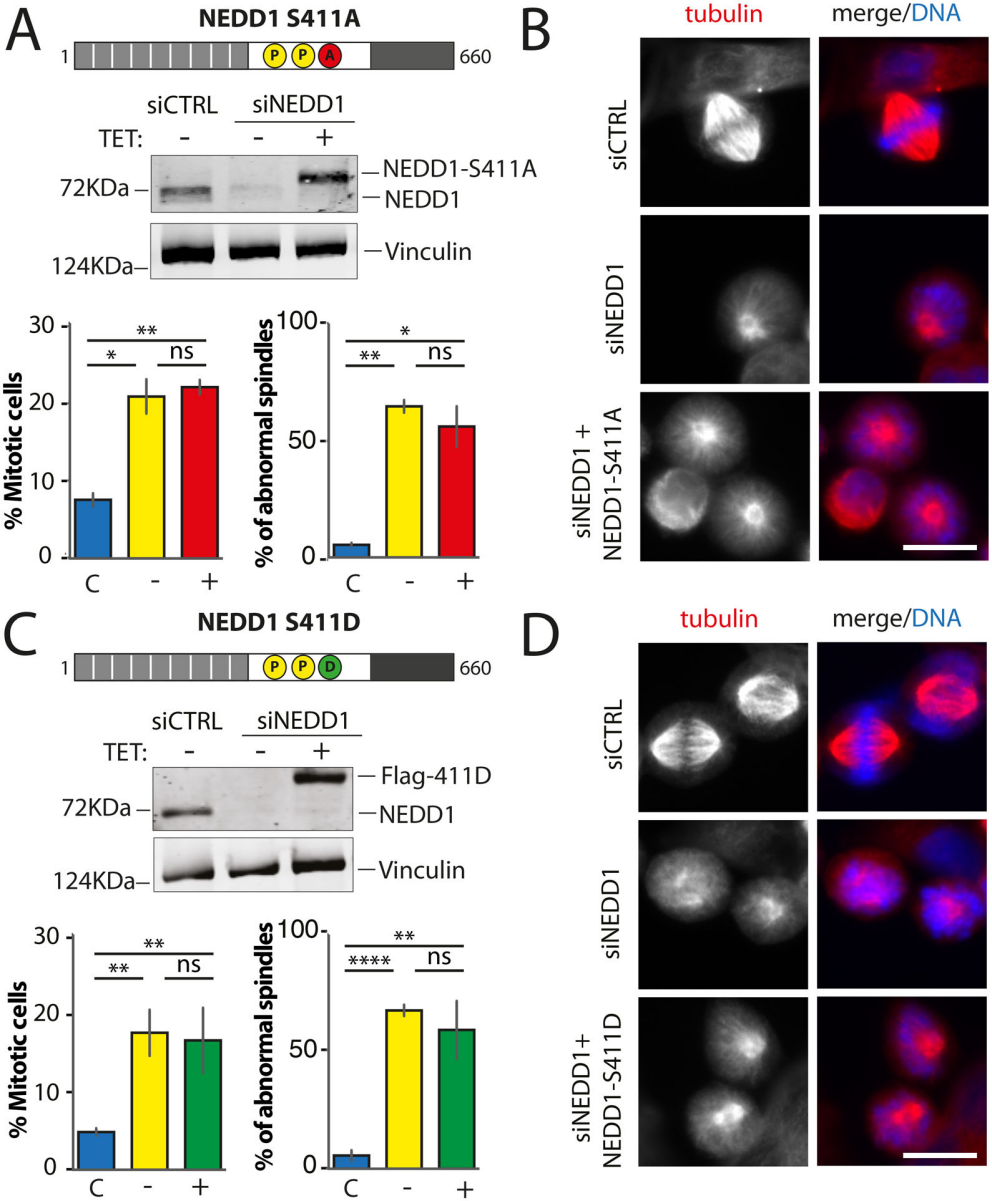
**Mitotic phenotypes in NEDD1 silenced HeLa cells upon expression of NEDD1 S411 phospho-variants.** (A,C) Upper panels: western blots showing NEDD1 protein levels in control (siCTRL) and NEDD1-silenced (siNEDD1) HeLa cells lines expressing or not Flag-NEDD1 phospho-variants at S411, as indicated (+TET). The anti-human NEDD1 antibody detects both the endogenous and ectopically expressed Flag-NEDD1 proteins. The vinculin band was used as a loading control. Lower panels: quantifications of the percentages of mitotic cells and abnormal spindles in HeLa cells in the different experimental conditions. Blue bars correspond to control cells not induced with tetracycline (C); yellow bars are NEDD1 silenced cells not induced with tetracycline (–); green and red bars correspond to NEDD1 silenced cells induced with tetracycline that express respectively the phospho-null Flag-NEDD1-S411A or phospho-mimetic Flag-NEDD1-S411D variants, as indicated (+). More than 1000 cells were monitored to calculate the percentage of mitotic cells and more than 200 to quantify the spindle phenotypes. The graphs represent the average from two (S411A) and three (S411D) independent experiments. Error bars are standard deviation. ns: not significant; *P<0,02; **P<0,01; ****P<0,0001. (B,D) Immunofluorescence of control (siCTRL) and NEDD1-silenced (siNEDD1) HeLa cells expressing Flag-NEDD1-S411A and Flag-NEDD1-S411D. DNA is in blue and tubulin is in red. Scale bar: 10 µm.

Previous studies showed that NEDD1-S377 phosphorylation is required for γ-tubulin recruitment to the centrosomes in prometaphase (Sdelci et al., 2012) but the consequences on bipolar spindle assembly had not been described. Expression of Flag-NEDD1-S377A in silenced cells did restore the assembly of MT arrays, consistently reduced in silenced mitotic cells (Figs 1–5). However, it did not restore spindle assembly nor progression through mitosis (Fig. 3A and B). These data suggest that Flag-NEDD1-S377A supports chromosome-dependent MT assembly but these MTs cannot form a bipolar spindle under these conditions. Consistently, expression of Flag-NEDD1-S377D that was previously shown to rescue γ-tubulin recruitment to the centrosome in prometaphase did also rescue spindle assembly in silenced cells (Sdelci et al., 2012) (Fig. 3C and D). Altogether we conclude that NEDD1 phosphorylation at S377 that plays an essential role in centrosome maturation and MT nucleation at the centrosomes also provides the required balance between the centrosomal and acentrosomal MTs pathways required for spindle assembly.

We previously identified and characterized the role of NEDD1 phosphorylation at S405 and showed that it is essential for chromosome/RanGTP-dependent MT nucleation and spindle assembly in HeLa cells and Xenopus egg extracts (Pinyol et al., 2013; Scrofani et al., 2015). Here, we obtained consistent results using HeLa-stable inducible cell lines. The expression of Flag-NEDD1-S405A in silenced cells did not rescue spindle assembly nor mitotic progression (Fig. 4A and B). In contrast, expression of Flag-NEDD1-S405D in silenced cells efficiently rescued spindle assembly and mitotic progression (Fig. 4C and D). These data further confirmed that RanGTP/chromosome-dependent MT nucleation has an essential role in spindle assembly in mammalian cells (Meunier and Vernos, 2016).

Altogether, our data suggest a key role for both the centrosomal and chromosome-dependent MTs for spindle assembly and mitotic progression in mammalian cells. They confirm key regulatory functions for NEDD1 phosphorylation for the activity of these two pathways in mitotic cells.

### NEDD1 phosphorylation at S411 plays a critical role during spindle assembly in mitotic cells

We went on to characterize NEDD1 S411 phospho-variants. Using a phospho-null variant of NEDD1-S411 previous studies showed that the phosphorylation at this site was required for MT branching and amplification (Lüders et al., 2006) and for spindle assembly (Johmura et al., 2011). Consistently, we found that expression of Flag-NEDD1-S411A in silenced cells did not rescue spindle assembly nor mitotic progression (Fig. 5A and B). Since any potential rescuing activity of the phospho-mimetic variant on S411 had not been addressed so far, we then explored whether expression of Flag-NEDD1-S411D could rescue spindle assembly in silenced cells. Strikingly, we found that it did not rescue spindle assembly nor mitotic progression (Fig. 5C, 5D and Fig. S1A). In fact, the mitotic phenotypes in silenced cells expressing this variant were very similar and reminiscent of those of silenced cells indicating a null function for this phospho-mimetic variant (Fig. 5D and Fig. S1B). As a first attempt to rule out any potential deleterious effect on the protein function owing to the introduction of an aspartic residue at position 411, we performed additional rescue experiments using another phospho-mimetic variant, Flag-NEDD1-S411E. The mitotic phenotypes in silenced cells expressing this variant were very similar to those observed previously with the other phospho-mimetic variant as well as for silenced cells.

These results indicated a critical role for NEDD1-S411 phosphorylation in spindle assembly and suggested that the regulation of MT-dependent MT amplification is key for spindle assembly in mammalian cells. However, it was not possible at this point to rule out that the amino acid substitutions at S411 could have a deleterious effect on NEDD1 functionality *per se*.

### NEDD1 S411 phosphorylation is essential for RanGTP-dependent MT branching in Xenopus *laevis* egg extracts

**Fig. 6.**
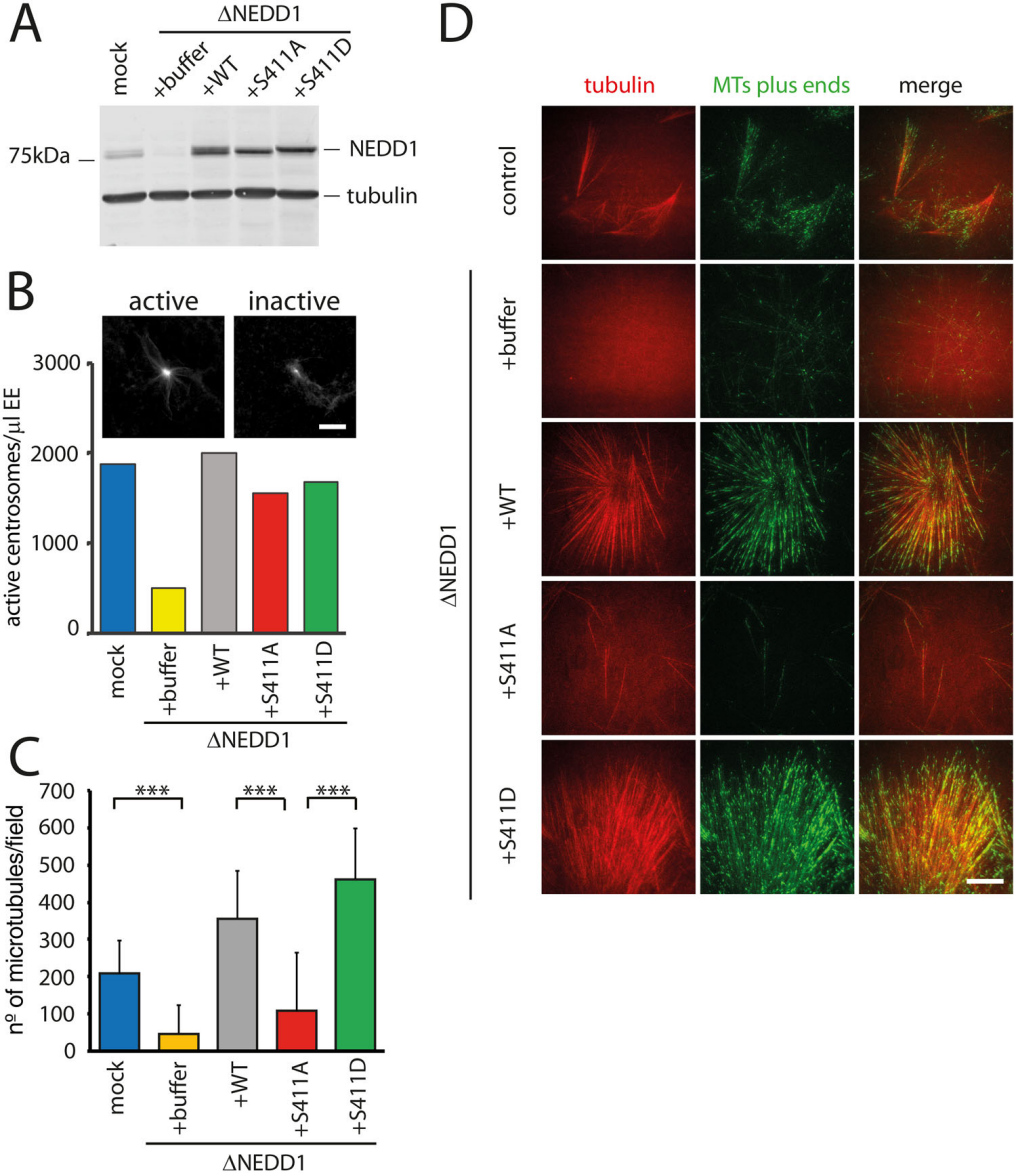
**Role of NEDD1 S411 phosphorylation in centrosomal and RanGTP-dependent MT nucleation in Xenopus *laevis* egg extracts.** (A) Western blots of control (mock) and NEDD1-depleted (ΔNEDD1) Xenopus *laevis* egg extracts supplemented or not (+buffer) with purified recombinant human Flag-NEDD1 proteins, as indicated. The anti-human NEDD1 antibody recognizes both the endogenous and exogenously added NEDD1 proteins. Tubulin was used as a loading control. (B) Activity of NEDD1 phospho-variant at S411 on the MT nucleation activity of centrosomes incubated in egg extracts. Human purified centrosomes were incubated in control (mock) and NEDD1-depleted egg extracts complemented or not with the indicated NEDD1 proteins. The number of centrosomes nucleating MTs after 30 min of incubation was counted in 30 random fields. The graph shows the quantifications from one out of two independent experiments. The images at the top show the microtubules (visualized through rhodamine-tubulin fluorescence) around purified centrosomes representative for active and inactive centrosomes. Scale bar: 10 µm. (C) Quantification of MTs assembled in RanGTP-complemented egg extracts. Control (mock) and NEDD1-depleted egg extracts complemented or not with the different human Flag-NEDD1 proteins (as indicated) were incubated for 20 min at 21°C and loaded into a flow chamber for TIRF imaging. Small amounts of Rhodamine labelled tubulin and GFP-Mal3 were added to respectively label the MTs and their growing plus-ends. The quantifications correspond to the number of MT plus ends in each field of view. Five random fields per condition in three independent experiments were analyzed. Error bars are standard deviations. ***P<0,001 (*t*-test). (D) Selected images from TIRF imaging of egg extracts in the different experimental conditions described and quantified in (C) Rhodamine tubulin (red) labels the MTs. GFP-Mal3 (green) labels the growing MTs plus-ends. Scale bar: 10 µm.

To address directly the functionality of the NEDD1-S411 phospho-variants we performed depletion and add-back experiments using the Xenopus *laevis* egg extract system. As previously described, NEDD1 was efficiently depleted from M-phase egg extracts (Fig. 6A). Purified Flag-NEDD1, Flag-NEDD1-S411A or Flag-NEDD1-S411D were added to the depleted extracts at close to endogenous concentrations (Fig. 6A) (Scrofani et al., 2015). We first checked whether NEDD1-S411 phosphorylation variants could restore centrosome-dependent MT nucleation in depleted extracts. Purified centrosomes were incubated in NEDD1-depleted egg extracts supplemented or not with Flag-NEDD1, Flag-NEDD1-S411A or Flag-NEDD1-S411D. The quantification of the centrosome associated MT asters in each condition showed that both NEDD1-S411 phosphorylation variants were as efficient as the wild type protein to restore MT assembly from centrosomes in depleted extracts (Fig. 6B). These results indicated that the phosphorylation of NEDD1 on S411 has no role in MT nucleation at the centrosome, consistently with previous reports in human cells (Lüders et al., 2006). They also indicated that the substitution of S411 by an aspartic residue has no deleterious consequences on NEDD1 functionality.

We then addressed directly the role of NEDD1-S411 phosphorylation in MT branching induced by RanGTP in egg extracts by TIRF microscopy. In control extracts, robust MT nucleation and branching could be observed, as previously described (Fig. 6C and D) (Petry et al., 2013). Instead, no or very few MTs with no branches could be observed in NEDD1-depleted extracts under similar conditions. Addition of Flag-NEDD1 to the depleted extract fully restored MT nucleation and branching (Fig. 6C and D) but not addition of Flag-NEDD1-S411A. In contrast, Flag-NEDD1-S411D was as efficient as Flag-NEDD1 for restoring MT nucleation and branching in depleted extracts (Fig. 6C and D).

These results show directly for the first time that NEDD1-S411 phosphorylation is essential for RanGTP-chromosome-dependent MT nucleation and branching. They also show that introducing an aspartic residue at position 411 does not interfere with NEDD1 activity. Indeed, Flag-NEDD1-S411D supports as efficiently as the wild type protein both centrosomal and non centrosomal MT nucleation and branching in Xenopus egg extracts.

### NEDD1-S411 phosphorylation promotes spindle bipolarization in Xenopus *laevis* egg extracts

**Fig. 7.**
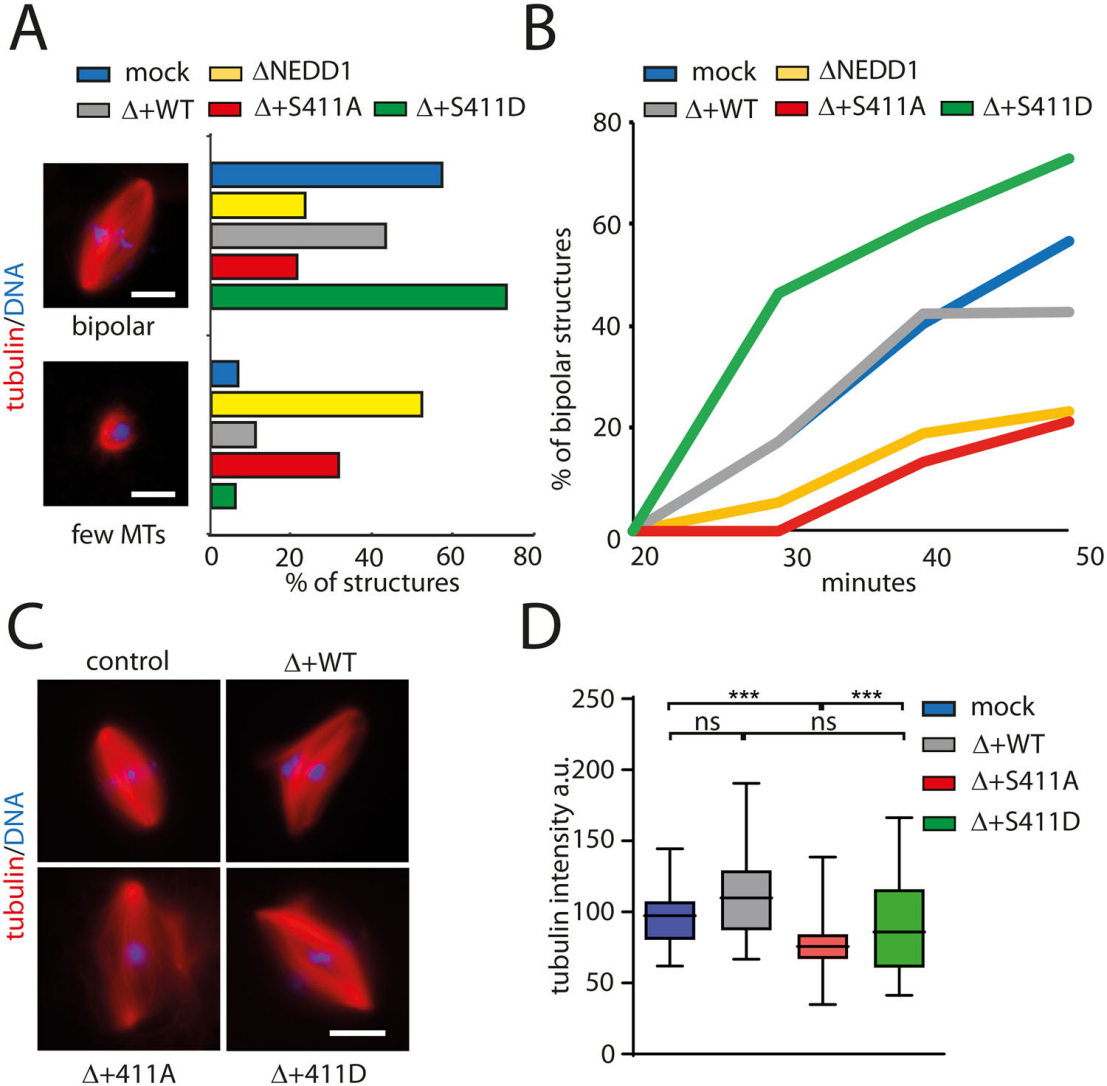
**Role of NEDD1 S411 phosphorylation in spindle assembly in Xenopus *laevis* egg extracts.** (A) Quantification of spindle structures assembled around sperm nuclei in cycled control (mock) and NEDD1-depleted egg extracts complemented or not with purified human Flag-NEDD1 variants as indicated. Representative images of a bipolar spindle (bipolar) and an aberrant structure typically found in NEDD1-depleted extract are shown (few MTs). Rhodamine labelled MTs (red) and DNA (blue). Scale bar: 10 μm. The graphs represent the percentage of the two types of structures in the different experimental conditions as indicated. More than 50 spindles were counted for each experimental condition. One representative out of four independent experiments with similar distributions. Scale bars: 10 μm. (B) Time course analysis of spindle assembly in the same experimental conditions as in A. More than 50 spindles were counted for each time point and experimental condition. The graph corresponds to one representative experiment out of three independent experiments. (C) Representative images of spindles assembled in control (mock) and NEDD1-depleted egg extracts complemented or not with purified human Flag-NEDD1 variants, as indicated. Rhodamine labelled MTs (red) and DNA (blue). Scale bar: 10 μm. (D) Box-and-whisker plots showing MT density in bipolar spindles assembled in the same experimental conditions as in C. MT density is expressed as the total tubulin fluorescence intensity normalized for the spindle area. More than 20 spindles were measured for each condition. The graph corresponds to one representative experiment out of three independent experiments. ***P<0,001 (*t*-test).

Since we previously found that Flag-NEDD1-S411D does not support spindle assembly in silenced HeLa cells, we decided to explore its functionality in spindle assembly in egg extracts. Consistently with previous studies, bipolar spindle assembly was strongly impaired in NEDD1-depleted egg extracts (Liu and Wiese, 2008; Pinyol et al., 2013) in which a large proportion of nuclei were surrounded by a few disorganized MTs (Fig. 7A). Addition of Flag-NEDD1 to depleted extracts efficiently rescued bipolar spindle assembly (Fig. 7A). In contrast, addition of Flag-NEDD1-S411A to the depleted extracts did not restore bipolar spindle assembly (Fig. 7A). Moreover, the few spindles that formed under these conditions had a low MT density (Fig. 7C and D). These data confirmed the essential role of NEDD1-S411 phosphorylation in spindle assembly. Strikingly, addition of Flag-NEDD1-S411D to depleted extracts rescued bipolar spindle assembly as efficiently as the wild type protein (Fig. 7A) fully restoring spindle MT density to control levels (Fig. 7D). In addition, spindle bipolarization occurred earlier in the presence of Flag-NEDD1-S411D than in control extracts (Fig. 7B), suggesting that this phospho-mimetic variant promotes a small change in the kinetics of spindle assembly in egg extracts without generating spindle defects.

Altogether our results provide direct support for the role of NEDD1 phosphorylation at S411 in acentrosomal MT nucleation and amplification. They also show that the phospho-mimetic Flag-NEDD1-S411D rescues fully spindle assembly in NEDD1-depleted extracts. This is consistent with the strict requirement for chromosome-dependent MTs for meiotic spindle assembly occurring in the absence of centrosomes in frogs and humans. Interestingly, Flag-NEDD1-S411D promoted some changes in the kinetics of MT organization since spindle bipolarization occurred more rapidly than in control conditions.

## DISCUSSION

NEDD1 is not a core component of the γ-TuRC (Consolati et al., 2020; Kollman et al., 2011). However, it is essential for the key function of this complex in the different MT nucleation pathways that drive MT assembly from the centrosomes, around the chromosomes and on pre-existing MTs during mitosis (Lüders et al., 2006; Pinyol et al., 2013; Sdelci et al., 2012). Bipolar spindles assemble and segregate chromosomes in the absence of centrosomes during female meiosis in mammals. However, during mitosis the bipolar spindle assembles in the presence of two centrosomes that actively nucleate MTs and act as major MT organizing centers. MTs that are nucleated through the centrosomal pathway may therefore not be essential for spindle assembly but a coordination between the acentrosomal and centrosomal MT nucleation pathways that compete for limiting components may be essential when centrosomes are present (Cavazza et al., 2016). Here we exploited the specific requirements of NEDD1 phosphorylation at different sites for centrosomal and acentrosomal MT nucleation to explore further the mechanism that control and integrate these pathways during bipolar spindle assembly.

Our work confirms data from previous studies but also extends them by providing a full characterization of the role of NEDD1 phosphorylation at S377, S405 and S411 in bipolar spindle formation and mitotic progression in the same experimental system. Our results provide further support for the essential role of NEDD1 phosphorylation in controlling the centrosomal and chromosome/RanGTP-dependent MT assembly pathways during spindle assembly (Khodjakov et al., 2000; Lüders et al., 2006; Meunier and Vernos, 2011). Phosphorylation of NEDD1 S377 and S405 are strict requirements for centrosome and chromosome-dependent MT nucleation respectively. Since the respective phospho-mimetic mutants are effective in rescuing spindle assembly, it suggests that there are no specific requirements for a temporal regulation of phosphorylation at these two sites. Previous studies have shown that spindles assemble in human cells experimentally manipulated to eliminate the centrosomes and these cells can proliferate indefinitely in the absence of centrosomes (Lambrus et al., 2015). However, in these cells, spindles assemble at a slower rate and display chromosome alignment defects (Khodjakov et al., 2000; Lambrus et al., 2015; Wong et al., 2015). Expression of Flag-NEDD1-S377A did promote more dramatic defects in spindle formation and mitotic progression. In the Flag-NEDD1-S377A expressing cells, centrosomes are still present but their activity is strongly reduced as this phospho-null variant does not support the recruitment of γ-tubulin during centrosome maturation. Altogether, our data therefore suggest that the reduced MTOC activity of the centrosomes interferes with the organization of the chromosome-dependent MTs into a bipolar spindle, and points to the importance of a proper balance between the different MT assembly pathways in mitosis.

Interestingly, our data reveal a novel function for NEDD1-S411 phosphorylation that is essential for spindle assembly in cells containing centrosomes. We found that although NEDD1-S411D is a fully functional protein that supports both centrosomal and acentrosomal MT nucleation as well as spindle assembly in egg extracts, it fails to rescue spindle assembly in NEDD1 silenced HeLa cells. In fact, the associated mitotic phenotypes observed in NEDD1-S411D expressing cells are similar to those of NEDD1 silenced cells. These results suggest that in addition to the essential requirement of NEDD1 phosphorylation at S411 for MT-based MT nucleation, some additional regulation is required to allow spindle assembly in cells containing centrosomes. It is tempting to speculate that this regulation is important to coordinate the activities of the centrosomal and acentrosomal MT nucleation pathways. Our data also suggest a dominant role for the phospho-mimetic S411D substitution, since the phospho-mimetic substitutions of S377 and S405 that are functional individually are not able to drive spindle assembly when in combination with S411D.

**Fig. 8.**
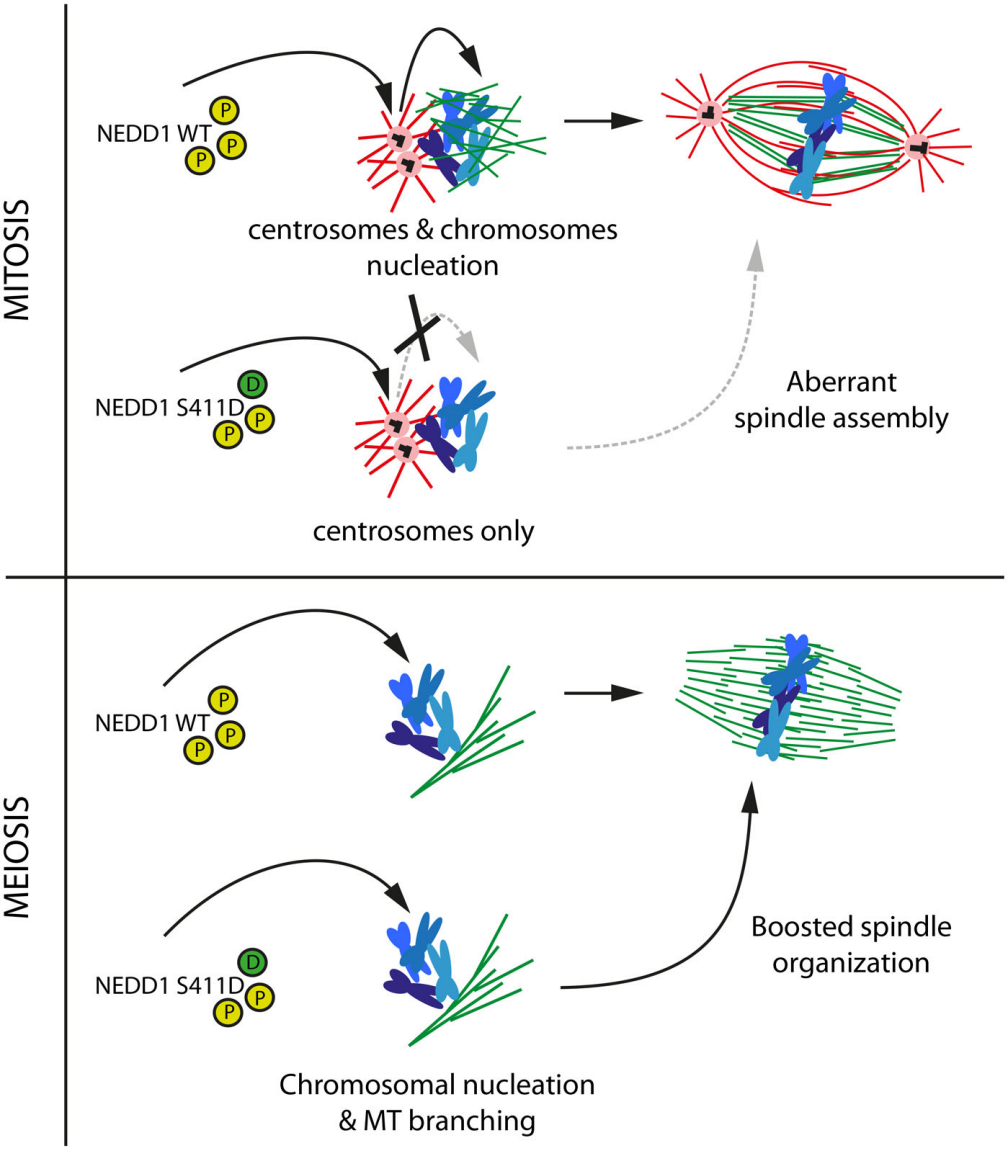
**Model: Cdk1 phosphorylation on NEDD1 S411 is a central and critical event for spindle assembly in mitosis and meiosis**. A precise regulation of NEDD1 S377, S405 and S411 phospho-events (yellow circles) define the equilibrium between MT nucleation from centrosomes (red) and chromosomes (green). The phospho-variant NEDD1 S411D that mimics a constitutively phosphorylated state (green circle) is detrimental for spindle assembly in HeLa cells but functional in Xenopus *laevis* egg extract. Upper panel: NEDD1-S411D may be retained at the centrosome impairing its function in the chromosome-dependent MT nucleation pathway and thereby spindle assembly. Lower panel: instead, in egg extracts, it promotes chromosome-dependent MT nucleation and branching and the fast organization of the bipolar spindle.

Mechanistically we envisage two possible scenarios to explain the different impact of NEDD1-S441D on spindle assembly in egg extracts and HeLa cells. Our data in egg extracts showed that NEDD1-S411D promotes the organization of the chromosomal MTs detected by the faster kinetics of bipolar spindle assembly. This change in spindle assembly kinetics may be well tolerated for the large meiotic spindle. In these spindles MT organization principles differ from those of smaller spindles assembled in somatic cells containing centrosomes (Brugués et al., 2012; O'Toole et al., 2020). However, in cells containing centrosomes, a change in the chromosomal MT organization kinetics could interfere with the correct and necessary integration of these MTs with those nucleated at the centrosomes that act as major MTOCs. Another possibility is that NEDD1-S411D localization dynamics is altered in cells containing centrosomes (Fig. 8). We observed that NEDD1-S411D localizes to the centrosome (data not shown). If the turnover of the protein at the centrosome is altered, it may not be accessible to perform its function in chromosome-dependent MT nucleation and branching. In agreement with this hypothesis, we did not observe many MTs in mitotic cells expressing these phospho-variants although these data need to be confirmed.

Altogether, our work highlights differential requirements for spindle assembly in meiotic and mitotic systems in which NEDD1 phosphorylation at S411 plays a key role. They suggest the existence of a mechanism for the coordination of MT nucleation through the centrosomal and acentrosomal pathways that is essential for ensuring spindle assembly in somatic cells containing centrosomes.

## MATERIALS AND METHODS

### Antibodies

The anti-NEDD1 antibody (Abnova M05) was used at 1:300 in immunofluorescence and at 1:600 in western-blot. The anti-Flag antibody (Sigma, F1804) was used 1:1000 in western-blot. Vinculin antibody (Sigma, V9131) was used at 1:20,000 in western-blot. The anti β-tubulin (Abcam ab6046) was used 1:300 for immunofluorescence. The anti DM1A tubulin (Sigma, T6557) was used 1:10,000 in western-blot. The anti Xenopus NEDD1 was raised against the C-terminal of the protein as described before (Pinyol et al., 2013). Secondary antibodies were anti-rabbit and anti-mouse antibodies conjugated to Alexa-488 or 568, 680 or 800 (Molecular Probes) and were used at 1:1000 for immunofluorescence and 1:10,000 for western blotting. Hoechst 3342 (Invitrogen, H3570) was used 1 µg/ml for immunofluorescence.

### Cell culture and stable cell lines

To generate cell lines expressing NEDD1 WT and the different NEDD1 phospho-variants, a siRNA-resistant ORF of NEDD1 was cloned in pcDNA5/FRT/TO (Invitrogen). Mutations of S377, S405 and S411 in Alanine and in Aspartic/Glutamic acids were then generated by site directed mutagenesis. These plasmids were transfected into a HeLa-FRT cell line (gift from Jonathon Pines, Institute of Cancer Research, London, UK) and stable cell lines were generated using the FLIP-in system (Invitrogen). Plasmids were transfected with X-tremeGENE 9 (Roche) following manufacturer's protocol. Positive clones were selected using hygromycin B at 0.6 mg/ml (Invitrogen) and picked up using cloning cylinders or cloning discs (Sigma). To induce protein expression from the inducible promoter, cells were incubated with tetracycline (Calbiochem) at 0.05 µg/ml. All cells were grown in DMEM, 10% fetal calf serum and penicillin/streptomycin (Life Technologies) and kept at 37°C and 5% CO_2_ humid atmosphere.

### Silencing and rescue experiments

All NEDD1 mutants were made on the NM_152905 sequence [lacking 7 amino acids in the N-terminal respect the isoform of NM_001135175 corresponding to GCP-WD (Lüders et al., 2006)]. To perform rescue experiments, expression of NEDD1 WT was induced the day before siRNA transfection and the media was changed every 24 h to keep the tetracycline concentration constant. Cells were silenced for a total of 55 h while NEDD1-WT was expressed during 72 h. siRNA targeting NEDD1 and scrambled siRNA were previously described (Lüders et al., 2006). They were transfected with Lipofectamine RNAiMAX (Invitrogen) using 100 pmols per well in six-well plates according to the manufacturer's protocol and analyzed 55 h after transfection.

### Immunofluorescence, microscopy and imaging

Cells were grown on coverslips and were fixed in −20°C methanol for 10 min. Blocking and antibody dilution buffer was 0.5% BSA (Sigma), 0.1% TritonX100 (Sigma). Fixed cells were visualized with an X63 objective on an inverted DMI-6000 Leica wide-field fluorescent microscope. Sample preparation for TIRF imaging on egg extract was performed as described previously (Petry et al., 2013). Imaging was performed on a Leica DMI-6000 GSD with a X100 TIRF objective. Pictures were acquired with the Leica Application Suite software. Images were processed with Image J or Photoshop (Adobe) and mounted in figures using Illustrator (Adobe).

### Cell lysates preparation, SDS-PAGE and western blots

To prepare lysates cells pellets were incubated in lysis buffer 30 min on ice (20 mM Tris-HCl pH 7.5-8, 137 mM NaCl, 1 mM EDTA, 1.5 mM MgCl_2_, 10% glycerol, 1% TritonX100). Lysates were then clarified by centrifugation 15 min at 13,200 rpm and 4°C on a table top centrifuge. Protein concentration was measured using the Bradford protein assay (Bio-Rad). When indicated, cell lysates were supplemented with Lambda Protein Phosphatase (P0753S, NEB) at a concentration of 200 units/50 μl reaction during 30 min at 30°C. For western blots, 30 to 40 μg of protein extract were loaded per lane for SDS-PAGE (8% acrylamide concentration). Coomassie staining was performed using BlueSafe staining (MB15201, NZYTECH). All gels were analyzed with the Odyssey Infrared imaging system (Li-Cor).

### Xenopus *laevis* egg extract

All work involving animals was done according to standard protocols approved by the Centre for Genomic Regulation Ethics Committee. Cytostatic-factor-arrested egg extracts from Xenopus *laevis* and cycled egg extract spindle assembly reactions were prepared as previously described (Desai et al., 1998; Peset et al., 2005). Centrosomal MTs assembly reactions and spindle assembly reactions around sperm nuclei were performed as previously described (Peset et al., 2005). RanGTP branched MTs assembly was performed as previously described (Petry et al., 2013). Reactions were incubated 20 min at 21°C and then flowed into a 10 µl chamber. A minimum of five images per condition were then randomly acquired. GFP-Mal3 comets were background subtracted, thresholded and counted automatically using the ‘analyze particle’ tool on Fiji (Rasband, W.S., ImageJ, U. S. National Institutes of Health, Bethesda, MD, USA, https://imagej.nih.gov/ij/).

Human FlagNEDD1-WT, -S411A and −411D were expressed and purified as previously described (Pinyol et al., 2013). Recombinant RanQ69 L (RanGTP) was expressed and purified as previously described (Brunet et al., 2004). Mal3-GFP was expressed and purified as previously described (Bieling et al., 2007).

Plasmids and cell lines are available upon request.

## Supplementary Material

10.1242/biolopen.059474_sup1 Supplementary information

## References

[BIO059474C1] Bieling, P., Laan, L., Schek, H., Munteanu, E. L., Sandblad, L., Dogterom, M., Brunner, D. and Surrey, T. (2007). Reconstitution of a microtubule plus-end tracking system in vitro. *Nature* 450, 1100-1105. 10.1038/nature06386 18059460

[BIO059474C2] Brugués, J., Nuzzo, V., Mazur, E. and Needleman, D. J. (2012). Nucleation and transport organize microtubules in metaphase spindles. *Cell* 149, 554-564. 10.1016/j.cell.2012.03.027 22541427

[BIO059474C3] Brunet, S., Sardon, T., Zimmerman, T., Wittmann, T., Pepperkok, R., Karsenti, E. and Vernos, I. (2004). Characterization of the TPX2 domains involved in microtubule nucleation and spindle assembly in Xenopus egg extracts. *Mol. Biol. Cell* 15, 5318-5328. 10.1091/mbc.e04-05-0385 15385625 PMC532013

[BIO059474C4] Cavazza, T., Malgaretti, P. and Vernos, I. (2016). The sequential activation of the mitotic microtubule assembly pathways favors bipolar spindle formation. *Mol. Biol. Cell* 27, 2935-2945. 10.1091/mbc.E16-05-0322 27489339 PMC5042580

[BIO059474C5] Clarke, P. R. and Zhang, C. (2008). Spatial and temporal coordination of mitosis by Ran GTPase. *Nat. Rev. Mol. Cell Biol.* 9, 464-477. 10.1038/nrm2410 18478030

[BIO059474C6] Clausen, T. and Ribbeck, K. (2007). Self-organization of anastral spindles by synergy of dynamic instability, autocatalytic microtubule production, and a spatial signaling gradient. *PLoS One* 2, e244. 10.1371/journal.pone.0000244 17330139 PMC1797610

[BIO059474C7] Consolati, T., Locke, J., Roostalu, J., Chen, Z. A., Gannon, J., Asthana, J., Lim, W. M., Martino, F., Cvetkovic, M. A., Rappsilber, J. et al. (2020). Microtubule nucleation properties of single human γTuRCs explained by their Cryo-EM structure. *Dev. Cell* 53, 603-617.e8. 10.1016/j.devcel.2020.04.019 32433913 PMC7280788

[BIO059474C8] Desai, A., Murray, A., Mitchison, T. J. and Walczak, C. E. (1998). The use of Xenopus egg extracts to study mitotic spindle assembly and function in vitro. *Methods Cell Biol.* 61, 385-412. 10.1016/S0091-679X(08)61991-3 9891325

[BIO059474C9] Gomez-Ferreria, M. A., Bashkurov, M., Helbig, A. O., Larsen, B., Pawson, T., Gingras, A. C. and Pelletier, L. (2012). Novel NEDD1 phosphorylation sites regulate gamma-tubulin binding and mitotic spindle assembly. *J. Cell Sci.* 125, 3745-3751. 10.1242/jcs.105130 22595525

[BIO059474C10] Goshima, G., Mayer, M., Zhang, N., Stuurman, N. and Vale, R. D. (2008). Augmin: a protein complex required for centrosome-independent microtubule generation within the spindle. *J. Cell Biol.* 181, 421-429. 10.1083/jcb.200711053 18443220 PMC2364697

[BIO059474C11] Gunawardane, R. N., Martin, O. C. and Zheng, Y. (2003). Characterization of a new γTuRC subunit with WD repeats. *Mol. Biol. Cell* 14, 1017-1026. 10.1091/mbc.e02-01-0034 12631720 PMC151576

[BIO059474C12] Haren, L., Stearns, T. and Lüders, J. (2009). Plk1-dependent recruitment of gamma-tubulin complexes to mitotic centrosomes involves multiple PCM components. *PLoS One* 4, e5976. 10.1371/journal.pone.0005976 19543530 PMC2695007

[BIO059474C13] Johmura, Y., Soung, N. K., Park, J. E., Yu, L. R., Zhou, M., Bang, J. K., Kim, B. Y., Veenstra, T. D., Erikson, R. L. and Lee, K. S. (2011). Regulation of microtubule-based microtubule nucleation by mammalian polo-like kinase 1. *Proc. Natl. Acad. Sci. USA* 108, 11446-11451. 10.1073/pnas.1106223108 21690413 PMC3136274

[BIO059474C14] Khodjakov, A., Cole, R. W., Oakley, B. R. and Rieder, C. L. (2000). Centrosome-independent mitotic spindle formation in vertebrates. *Curr. Biol.* 10, 59-67. 10.1016/S0960-9822(99)00276-6 10662665

[BIO059474C15] Kollman, J. M., Merdes, A., Mourey, L. and Agard, D. A. (2011). Microtubule nucleation by gamma-tubulin complexes. *Nat. Rev. Mol. Cell Biol.* 12, 709-721. 10.1038/nrm3209 21993292 PMC7183383

[BIO059474C16] Lambrus, B. G., Uetake, Y., Clutario, K. M., Daggubati, V., Snyder, M., Sluder, G. and Holland, A. J. (2015). p53 protects against genome instability following centriole duplication failure. *J. Cell Biol.* 210, 63-77. 10.1083/jcb.201502089 26150389 PMC4494000

[BIO059474C17] Liu, L. and Wiese, C. (2008). Xenopus NEDD1 is required for microtubule organization in Xenopus egg extracts. *J. Cell Sci.* 121, 578-589. 10.1242/jcs.018937 18252801

[BIO059474C18] Lüders, J., Patel, U. K. and Stearns, T. (2006). GCP-WD is a γ-tubulin targeting factor required for centrosomal and chromatin-mediated microtubule nucleation. *Nat. Cell Biol.* 8, 137-147. 10.1038/ncb1349 16378099

[BIO059474C19] Mahoney, N. M., Goshima, G., Douglass, A. D. and Vale, R. D. (2006). Making microtubules and mitotic spindles in cells without functional centrosomes. *Curr. Biol.* 16, 564-569. 10.1016/j.cub.2006.01.053 16546079

[BIO059474C20] Manning, J. A., Shalini, S., Risk, J. M., Day, C. L. and Kumar, S. (2010). A direct interaction with NEDD1 regulates γ-tubulin recruitment to the centrosome. *PLoS One* 5, e9618. 10.1371/journal.pone.0009618 20224777 PMC2835750

[BIO059474C21] Meunier, S. and Vernos, I. (2011). K-fibre minus ends are stabilized by a RanGTP-dependent mechanism essential for functional spindle assembly. *Nat. Cell Biol.* 13, 1406-1414. 10.1038/ncb2372 22081094

[BIO059474C22] Meunier, S. and Vernos, I. (2012). Microtubule assembly during mitosis - from distinct origins to distinct functions? *J. Cell Sci.* 125, 2805-2814. 10.1242/jcs.092429 22736044

[BIO059474C23] Meunier, S. and Vernos, I. (2016). Acentrosomal microtubule assembly in mitosis: the where, when, and how. *Trends Cell Biol.* 26, 80-87. 10.1016/j.tcb.2015.09.001 26475655

[BIO059474C24] O'Toole, E., Morphew, M. and Mcintosh, J. R. (2020). Electron tomography reveals aspects of spindle structure important for mechanical stability at metaphase. *Mol. Biol. Cell* 31, 184-195. 10.1091/mbc.E19-07-0405 31825721 PMC7001478

[BIO059474C25] Peset, I., Seiler, J., Sardon, T., Bejarano, L. A., Rybina, S. and Vernos, I. (2005). Function and regulation of Maskin, a TACC family protein, in microtubule growth during mitosis. *J. Cell Biol.* 170, 1057-1066. 10.1083/jcb.200504037 16172207 PMC2171525

[BIO059474C26] Petry, S., Groen, A. C., Ishihara, K., Mitchison, T. J. and Vale, R. D. (2013). Branching microtubule nucleation in Xenopus egg extracts mediated by augmin and TPX2. *Cell* 152, 768-777. 10.1016/j.cell.2012.12.044 23415226 PMC3680348

[BIO059474C27] Piehl, M., Tulu, U. S., Wadsworth, P. and Cassimeris, L. (2004). Centrosome maturation: measurement of microtubule nucleation throughout the cell cycle by using GFP-tagged EB1. *Proc. Natl. Acad. Sci. USA* 101, 1584-1588. 10.1073/pnas.0308205100 14747658 PMC341778

[BIO059474C28] Pinyol, R., Scrofani, J. and Vernos, I. (2013). The role of NEDD1 phosphorylation by Aurora A in chromosomal microtubule nucleation and spindle function. *Curr. Biol.* 23, 143-149. 10.1016/j.cub.2012.11.046 23273898

[BIO059474C29] Scrofani, J., Sardon, T., Meunier, S. and Vernos, I. (2015). Microtubule nucleation in mitosis by a RanGTP-dependent protein complex. *Curr. Biol.* 25, 131-140. 10.1016/j.cub.2014.11.025 25532896

[BIO059474C30] Sdelci, S., Schutz, M., Pinyol, R., Bertran, M. T., Regue, L., Caelles, C., Vernos, I. and Roig, J. (2012). Nek9 phosphorylation of NEDD1/GCP-WD contributes to Plk1 control of gamma-tubulin recruitment to the mitotic centrosome. *Curr. Biol.* 22, 1516-1523. 10.1016/j.cub.2012.06.027 22818914

[BIO059474C31] Wieczorek, M., Urnavicius, L., Ti, S. C., Molloy, K. R., Chait, B. T. and Kapoor, T. M. (2020). Asymmetric molecular architecture of the human gamma-tubulin ring complex. *Cell* 180, 165-175.e16. 10.1016/j.cell.2019.12.007 31862189 PMC7027161

[BIO059474C32] Wong, Y. L., Anzola, J. V., Davis, R. L., Yoon, M., Motamedi, A., Kroll, A., Seo, C. P., Hsia, J. E., Kim, S. K., Mitchell, J. W. et al. (2015). Cell biology. Reversible centriole depletion with an inhibitor of Polo-like kinase 4. *Science* 348, 1155-1160. 10.1126/science.aaa5111 25931445 PMC4764081

[BIO059474C33] Zhang, X., Chen, Q., Feng, J., Hou, J., Yang, F., Liu, J., Jiang, Q. and Zhang, C. (2009). Sequential phosphorylation of Nedd1 by Cdk1 and Plk1 is required for targeting of the gammaTuRC to the centrosome. *J. Cell Sci.* 122, 2240-2251. 10.1242/jcs.042747 19509060

[BIO059474C34] Zheng, Y., Wong, M. L., Alberts, B. and Mitchison, T. (1995). Nucleation of microtubule assembly by a γ-tubulin-containing ring complex. *Nature* 378, 578-583. 10.1038/378578a0 8524390

