# Supplementary material for: NEDD1-S411 phosphorylation plays a critical function in the coordination of microtubule nucleation during mitosis
Source: Biol Open. 2022 Nov 17;11(11):bio059474. doi: 10.1242/bio.059474 (PMC9836086; doi:10.1242/bio.059474)
Supplement: Supplementary information [file biolopen-11-059474-s1.pdf]

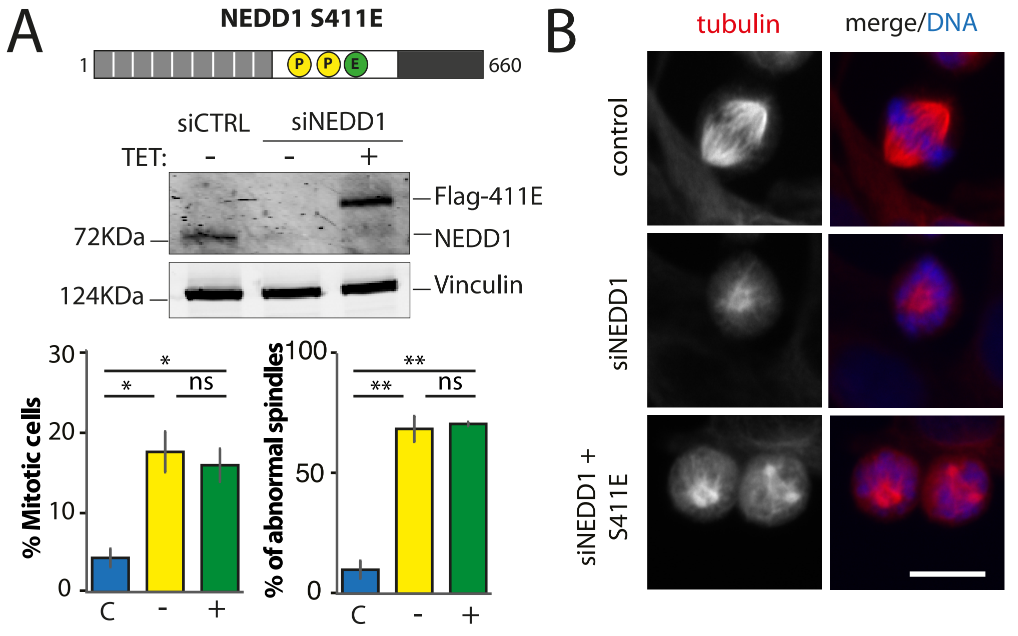

Figure S1

Fig. S1. A- Upper panel: Western-blot showing NEDD1 protein levels in control (siCTR) and NEDD1-silenced (siNEDD1) HeLa cells expressing Flag-NEDD1 phosphovariants (as indicated) under tetracycline control (+TET). The anti-human NEDD1 antibody detects both endogenous and ectopically expressed Flag-NEDD1. Vinculin was used as a loading control. Lower panel: Quantifications of the percentages of mitotic cells and abnormal spindles in HeLa cells in the different experimental conditions. Blue bars correspond to control cells not induced with tetracycline (C); yellow bars are NEDD1 silenced cells not induced with tetracycline (-); green and red bars correspond to NEDD1 silenced cells induced with tetracycline that express respectively the phospho-mimetic Flag-NEDD1-S411E variant (+). More than 1000 cells were monitored to calculate the percentage of mitotic cells and more than 200 to quantify the spindle phenotypes. The graphs correspond to the average from two independent experiments. Error bars are standard deviation. ns not significant; * p<0,03; **p<0,01. **B-** Immunofluorescence of control and NEDD1-silenced (siNEDD1) HeLa cell lines expressing Flag-NEDD1 S411E. DNA is in blue and tubulin is in red. Scale bar, 10 µm.

Biology Open • Supplementary information
